# Improved sensitivity and automation of a multi-step upconversion lateral flow immunoassay using a 3D-printed actuation mechanism

**DOI:** 10.1007/s00216-024-05156-5

**Published:** 2024-01-27

**Authors:** Kirsti Raiko, Oskari Nääjärvi, Miikka Ekman, Sonja Koskela, Tero Soukka, Iida Martiskainen, Teppo Salminen

**Affiliations:** Biotechnology Unit, Department of Life Technologies, Faculty of Technology, University of Turku, Kiinamyllynkatu 10, 20520 Turku, Finland

**Keywords:** Nanoparticles, Luminescence, Biomedical analysis, Bioassays, Polymers, Rare earth elements

## Abstract

The development of sensitive point-of-care (POC) assay platforms is of interest for reducing the cost and time of diagnostics. Lateral flow assays (LFAs) are the gold standard for POC systems, but their sensitivity as such is inadequate, for example, in the case of cardiac diagnostics. The performance can be improved by incorporating different steps, such as pre-incubation to prolong the interaction time between sample and reporter for immunocomplex formation, and washing steps for background reduction. However, for POC assays, manual steps by the assay conductor are not desired. In this research, upconverting nanoparticles (UCNPs) were coated with poly(acrylic acid) (PAA) and conjugated to anti-cTnI antibodies, yielding non-clustering particles with low non-specific binding. The performance of cTnI-LFA in the PAA-anti-cTnI-UCNPs was compared to the same UCNPs with a commercial carboxyl surface. A kitchen-timer mechanism was embedded in a 3D-printed housing to produce a low-cost actuator facilitating a timed pre-incubation step for reporter and sample, and a washing step, to enable a multi-step cTnI-LFA with minimized manual labour. PAA-UCNPs showed improved mobility on nitrocellulose compared to those with a commercial surface. The mechanical actuator system was shown to improve sensitivity compared to a labour-intensive multi-step dipstick method, despite pre-incubation occurring during shaking and heating in the dipstick method. The limit of detection decreased from 7.6 to 1.5 ng/L cTnI in human plasma. The presented actuator can be easily modified for sensitivity improvement in the LFA for different analytes via pre-incubation and washing steps.

**Graphical abstract:**

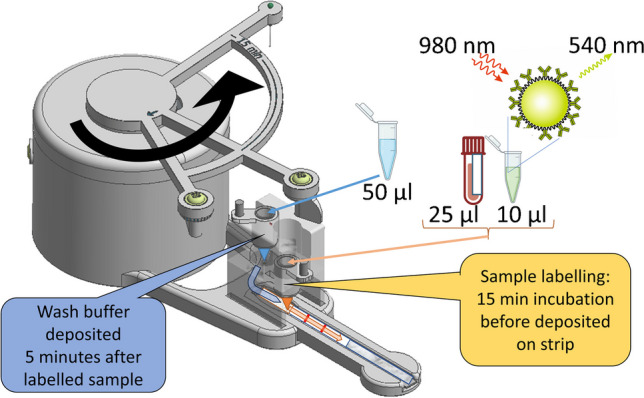

**Supplementary Information:**

The online version contains supplementary material available at 10.1007/s00216-024-05156-5.

## Introduction

Lateral flow assays (LFAs) and other paper-based diagnostic methods have been of interest in the diagnostic field for decades, although never as visibly as during the recent Sars-CoV-2 pandemic. They are inexpensive to manufacture and store and require little to no expertise to conduct. In short, LFAs are the closest to the gold standard in development of rapid, affordable, and easy-to-conduct point-of-care (POC) assays. The simplicity originates from the fact that they are often one-step, just add-the-sample assays requiring no special equipment or training, such as the basic home pregnancy test or Sars-COV-2 consumer tests. On the other hand, due to the same reason, they suffer from issues with sensitivity, making LFAs unsuitable for detection of analytes which have extremely low clinically relevant concentrations, requiring multi-step assays for reliable detection [1].

Incorporating additional washing steps to decrease background signal from the strip and controlling flow to enable a pre-incubation step to prolong interaction time between reporter and analyte for immunocomplex formation, have been key aspects of sensitivity improvement of POC-LFAs [2, 3]. To maintain user-friendliness, minimized required labour and costs, incorporation of these steps into the LF-strip is crucial. Thus, a variety of passive, strip-integrated, flow control methods have been developed to realize such multi-step assays [3, 4], ranging from diffusible sugar- or hydrophobic barriers [5, 6] and liquid path widening with absorbent pads [7], to wax pillars melted into the nitrocellulose structure [8] and complex networks printed with wax [9]. These approaches, however, are only capable of a few minute reproducible delays, as factors such as air humidity and temperature start to affect the rate of flow and evaporation from the porous assay strip. In addition, heavy modifications to the structure of mass-produced LF-strips tend to increase the price of the single-use test significantly. To automate a multi-step LFA, the liquid processing has been executed via mechanical kitchen timers. This enables reusable electricity free system with low cost and poses little to no modification needs to the LF strips themselves [10, 11].

Another way to improve the sensitivity of an LFA is to utilize light producing, optically read reporters instead of reporters read with a naked eye [12], enabling quantitative LFAs. Upconverting nanoparticles (UCNPs) are optically read reporters with unique capabilities for design of high-sensitivity assays. Unlike any other materials, such as those responsible for autofluorescence, UCNPs convert low-energy infrared-light to higher energy visible light [13]. This anti-Stokes process enables spectral elimination of autofluorescence background, and combined with the brightness and photostability of UCNPs, it facilitates extreme sensitivity of detection in immunoassays [14]. Feasibility and benefits of using UCNPs in LFAs (UCNP-LFAs) have been demonstrated in various applications, such as multiplex detection of water contaminants [15], COVID-19 diagnostics [16], detection of heat shock protein 70 [17], hepatitis B virus surface antigen [18], *Plasmodium falciparum* malaria [19], cardiac troponin I (cTnI) [20], and glycovariant identification on cancer biomarkers [21]. In many cases, clinically relevant sensitivities have been reached in these assays due to incorporation of upconverting reporters.

In previous research on UCNPs in immunoassays, it has been established that the surface chemistry of the used reporter particles plays an essential role in the performance of assays [14]. Surface chemistry affects the tendency of UCNPs to non-specifically bind to different surfaces and to each other (aggregation) [14, 22]. High non-specific binding of such extremely detectable reporters leads to higher background noise, and large size distribution of particle aggregates causes variation in results, both leading to decreased sensitivity [23].

In this research, cTnI is used as a model analyte in investigating the sensitivity improvement of the platform. In the event of cardiac damage, heart muscle disintegrates and cardiac proteins, including cTnI, are secreted into circulation [24]. Diagnosis of cardiac damage with cTnI is done by measuring the change of cTnI concentration in circulation within a certain time interval [25]. In cTnI detection, sensitivity is crucial as the smaller quantities of analyte the system can measure, the sooner the increase in cTnI can be detected. Desired limit of detection has been stated to be < 10 ng/L [26]. In addition to sensitivity of the assay, also speed and simplicity of the method is desired. Using a high-sensitivity POC-system, no sample transport between patient and central laboratory would be needed, reducing costs and waiting times. No validated high-sensitivity cTnI-POC is currently available, although a need for one has been expressed [27, 28].

**Fig. 1:**
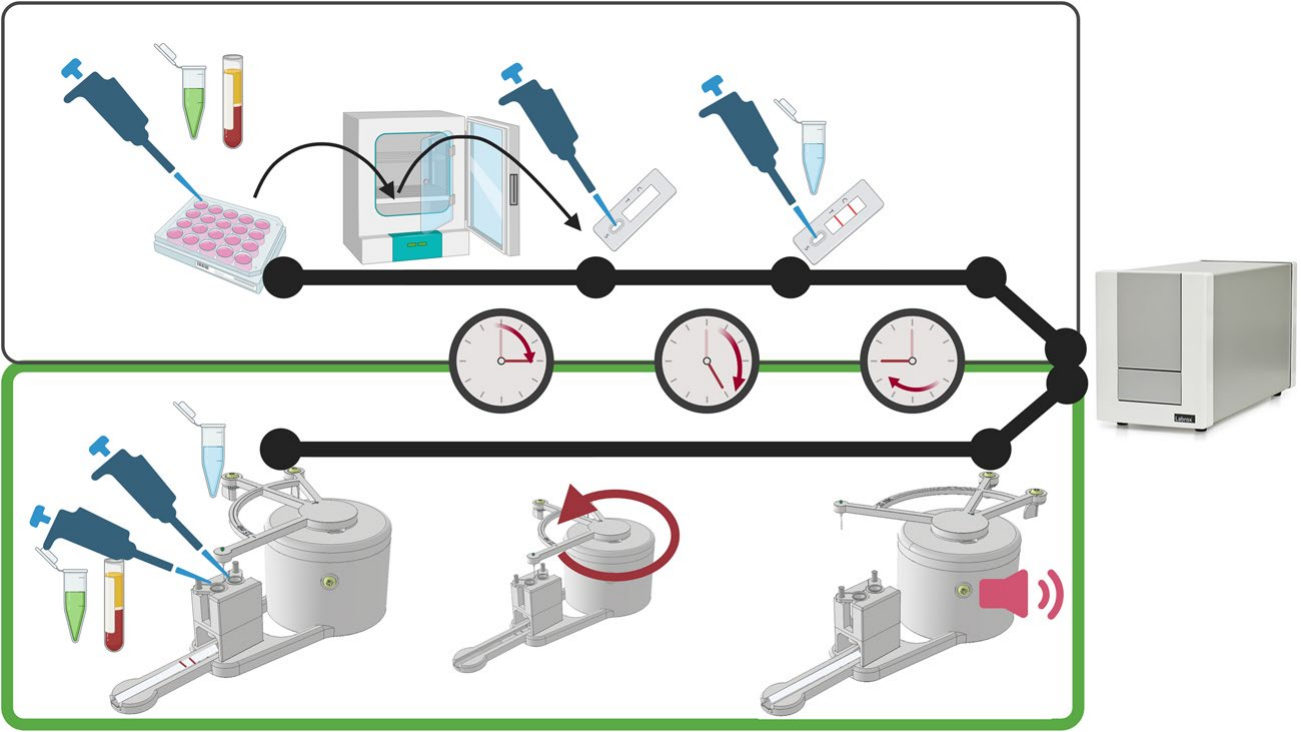
Difference in assay procedure with and without the mechanical control switch (below and above, respectively). With control switch system, the sample (plasma tube) and reporter (green tube) are pipetted together in the first deposition tube and wash buffer (blue tube) in the second tube. In the end when the bell rings, the strip is measured. Without the control switch, all these steps are manual, including timing. In addition, in the procedure above, the pre-incubation was done in a separate thermal shaker incubator. Dots along the black timeline represent manual steps, and the clocks show minutes passed since beginning of assay. Illustration created in BioRender.com

In previous published UCNP-LFA for cTnI [20], a 15-min heated pre-incubation of reporter and sample in shaking, as well as a separate washing step, were concluded to be necessary for sensitivity improvement in a cTnI-LFA. However, these modifications lead to a significant increase of manual labour and attention required of the assay conductor. In this research, the aim was to design an affordable mechanical device which enables a reproducible multi-step LFA without addition of manual labour or modifications to the LF-strip itself compared to the standard LF-procedures. The device utilizes the machinery of a classic mechanical kitchen timer, with a 3D-printed housing including a cassette for the LF-strip. The difference in assay procedure is highlighted in Fig. 1. A GIF-animation of the function is provided as Supplementary file 1 (ESM1).

Another aim of this research was to introduce a novel UCNP surface chemistry to an LF application to improve sensitivity through monodispersity and reduced non-specific binding of a highly detectable reporter.

## Materials and methods

### Materials and reagents 

**Fig. 2:**
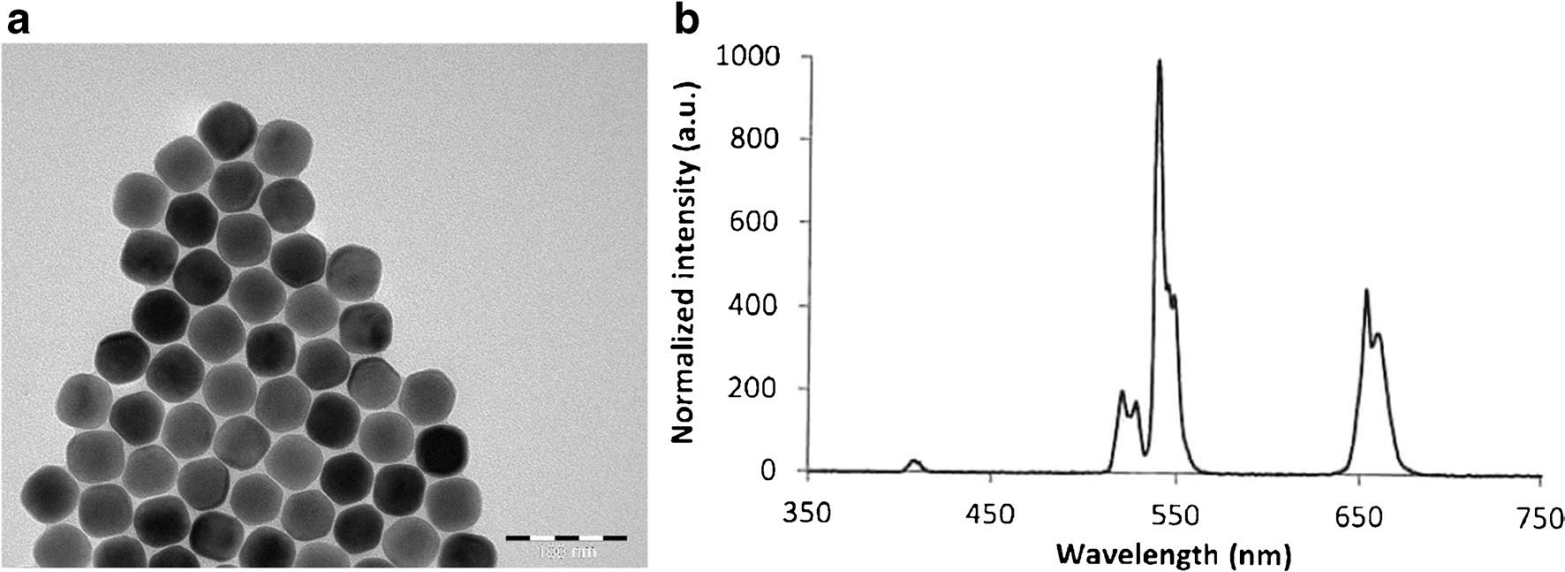
**a** Transmission electron microscopy image of non-functionalized, bare UCNP-cores, provided by Uniogen Oy. The size was declared to be 47 × 44 nm. The scale bar is 100 nm. **b** Emission spectrum of UCNPs under NIR-excitation (980 nm laser diode)

Monoclonal anti-human cTnI antibodies 916, 19C7cc, and 625, along with recombinant troponin ITC-complex, were purchased from Hytest Oy (Turku, Finland). Monoclonal antibody 9707 was purchased from Medix Biochemica (Espoo, Finland). Commercial carboxyl functionalized and non-functionalized NaYF_4_: Yb^3+^, Er^3+^-UCNP cores (RD Upcon®540-L-core) were obtained from Kaivogen Oy (Turku, Finland). Transmission electron microscopy image of non-functionalized UCNP cores, as well as the emission spectrum, were provided by Uniogen Oy and are shown in Fig. 2.

Non-functionalized UCNP-cores were coated with poly(acrylic acid) (PAA) (Sigma-Aldrich, Saint Louis, MO, USA) of Mw 2000 using a two-step ligand exchange method and bioconjugated with anti-cTnI Mab 625, following a previously published protocol [23] for both the coating and bioconjugation. For bioconjugation of commercially carboxylated UCNPs, the same conjugation protocol was followed.

Materials for lateral flow strips were Standard Grade Backing Laminate and KN-CPP1-Clear Kenosha cover plastic, both purchased from Kenosha Tapes (Amstelveen, Netherlands), nitrocellulose LFNC-C-BS023-70 (Nupore Filtration Systems Pvt. Ltd., Ghaziabad, India), glass fibre 8951 (Ahlstrom-Munksjö Oyj, Helsinki, Finland), and cellulose absorbent pad CFSP223000 by Merck Millipore (Burlington, MA, USA). 3D-printing filament (polylactic acid, PLA, filament width 1.75 mm) was purchased from Clas Ohlson (Insjön, Sweden). Mouse IgG was purchased from Meridian Bioscience Inc. (Cincinnati, OH, USA), while rabbit anti-mouse IgG was purchased from Invitrogen (Waltham, MA, USA). Denatured mouse IgG was prepared from mouse IgG via treatment in 63 °C for 30 min.

Plasma was collected from three apparently healthy volunteers, who provided informed consent, in lithium-heparin tubes (Vacuette® 9 ml, Greiner Bio-one, Kremsmünster, Austria) in compliance with the Helsinki agreement. Plasma was pooled, finalizing anonymity of donors, and aliquoted for storage in − 20 °C. Before use, it was thawed at + 22 °C and centrifuged at 3000 g for 10 min to remove denatured protein mass, and the supernatants cooled on ice were spiked with troponin ITC-complex to desired cTnI-concentrations.

All reagents not specified in this section were purchased from Sigma-Aldrich.

### Preparation of lateral flow strips

Sample pads (16 mm wide) were blocked by saturating with blocking buffer (10 mM borate buffer pH 7.5, 0.1% Tween-20, 0.5% BSA, 50 mM EDTA, 0.05% PAA) and dried for a minimum of 2 h in + 35 °C and used in the lateral flow test card assembly on backing support with nitrocellulose (25 mm wide) and absorbent pad (34 mm wide). Test lines (600 or 1000 ng/cm of Mab-19C7, Mab-916 and Mab-9707 in ratio 1:1:0.5) and control lines (600 or 1000 ng/cm RAM) in printing buffer (10 mM Tris pH 8, 5% EtOH, 1% Sucrose, 40 µg/ml cherry red) were dispensed onto nitrocellulose 10 mm from the front end of the membrane and 5 mm apart from each other, using Linomat 5 printer (Camag, Muttenz, Switzerland). After printing, the cards were allowed to dry overnight in + 35 °C, followed by addition of clear cover tape (34 mm wide), overlapping 1 mm with sample pad. Cards were stored in room temperature, protected from light and humidity, and cut to 4.8-mm-wide strips before use.

### Dipstick cTnI assay

The manual dipstick assay was based on a previously published protocol [20] and conducted to compare the performance of UCNPs with different surface chemistries and the actuator-mediated assay. Samples were prepared by spiking plasma with ITC-complex to standard concentrations of 0.5–500 ng/L. For pre-incubation, 25 µl of sample was pipetted on clear polystyrene microtiter plate, followed by 100 ng of Mab 625-conjugated reporter (commercially carboxylated UCNPs or PAA-UCNPs) in 25 µl of assay buffer (0.05 M Tris pH 7.5, 0.5 M NaCl, 0.04% NaN_3_, 2 mM KF, 1.5% BSA, 0.06% bovine gamma-globulin, 0.2 mg/ml mouse IgG, 0.05 mg/ml denatured mouse IgG, 0.05% PAA Mw 1200). Sample and reporter were pre-incubated usually for 15 min in 35 °C, 900 rpm shaking, apart from when effect of pre-incubation time to signal intensity was studied, in which case 5-, 7.5-, 10-, 12.5-, 15- and 20-min incubation times were used. After pre-incubation, the assay strips were dipped in the wells with reporter and sample, and the liquid was allowed to be absorbed for 9 min before initiation of wash step by moving the strip to another well containing 50 µl assay buffer. Strips were measured after a total time of 55 min after beginning of pre-incubation, using UPCON-reader (Labrox, Turku, Finland). The upconversion luminescence (UCL) signal from test and control line were scanned (125 scan points over the range of 25 mm covering the area of both test and control lines, 1 mm emission spot size, 100 ms measurement time) with 976 nm excitation laser using 100% relative laser power, reading emission at 540 nm.

### 3D-printing of actuator housing

All printed objects were designed and converted to. stl-files using the Computer Assisted Designing software Autodesk Inventor Professional 2021 (Autodesk Inc. Mill Valley, CA, USA). Designs were sliced for 3D-printing using the Prusa Slic3r Version 2.3.3. + win64 (Prusa Research by Josef Prusa) using default settings for 0.2 mm QUALITY profile for Generic PLA and 15% infill and printed out of PLA on Original Prusa i3 MK3S 3D-printer. The.stl-files are given as online resources (ESM2-ESM8) and a ready-to-print package of the actuator in 0.3mf file for Prusa printers as ESM9. In total, the mass of filament used was 82 g (according to PrusaSlicer program) and the printing time for all parts was approximately 9 h in total.

### Assembly of actuator

**Fig. 3:**
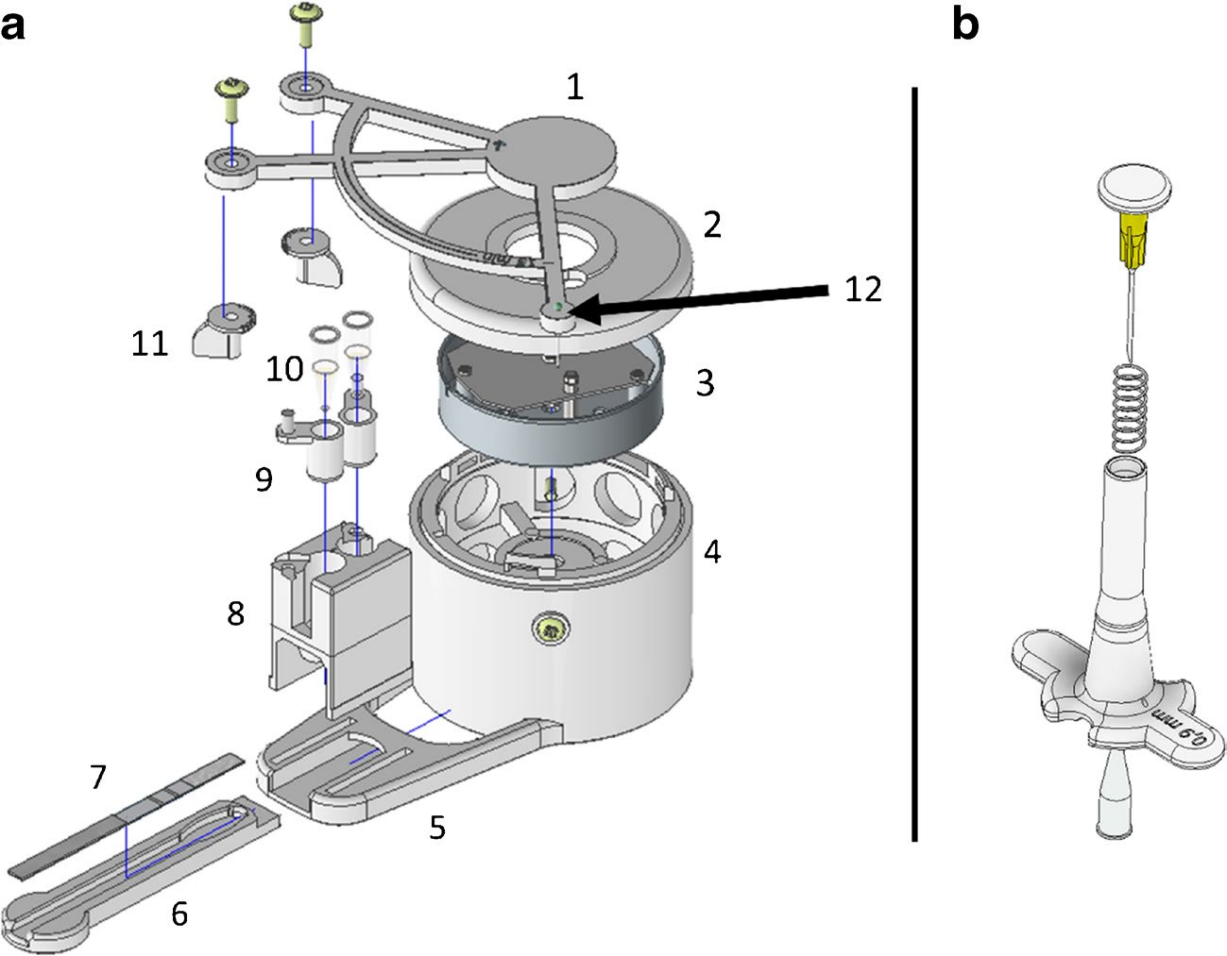
**a** Assembly diagram of the actuator. (1) Arm, (2) lid, (3) mechanical clockwork from kitchen timer, (4) body, (5) dock, (6) cassette, (7) LF-strip, (8) clutch, (9) tube holder, (10) deposition tubes with vents, (11) paw, (12) indicator pin. cTnI assay with actuator. **b** 3D-printed punching tool. The body is 3D-printed, after which a metal spring from a standard ball-point pen is inserted followed by a 0.9-mm injection needle attached to a 3D-printed head button. A PCR-tube is inserted bottom-up to be and the head button is pushed to puncture the bottom

Actuator was assembled from 3D-printed parts and a mechanism of a standard commercial mechanical kitchen timer (House, S Group, Helsinki, Finland) illustrated in Fig. 3. Paws were attached to the arm and the mechanical clockwork inside the body with metal screws. A dulled head pin was used as the indicator pin on the arm, indicating the exact position from which the first paw is 15 and the second 20 min away. Deposition vents were punctured to the bottoms of PCR tubes (V = 200 µl) with lids removed, using a 0.9-mm-needle integrated to a 3D-printed punching tool (Fig. 3b). The associated.stl-files given as supplementary files ESM10-ESM11. The punching tool ensures replicable punching always in the same position and orientation and minimizes the risk of physical injury.

### Actuator mediated assay

**Fig. 4:**
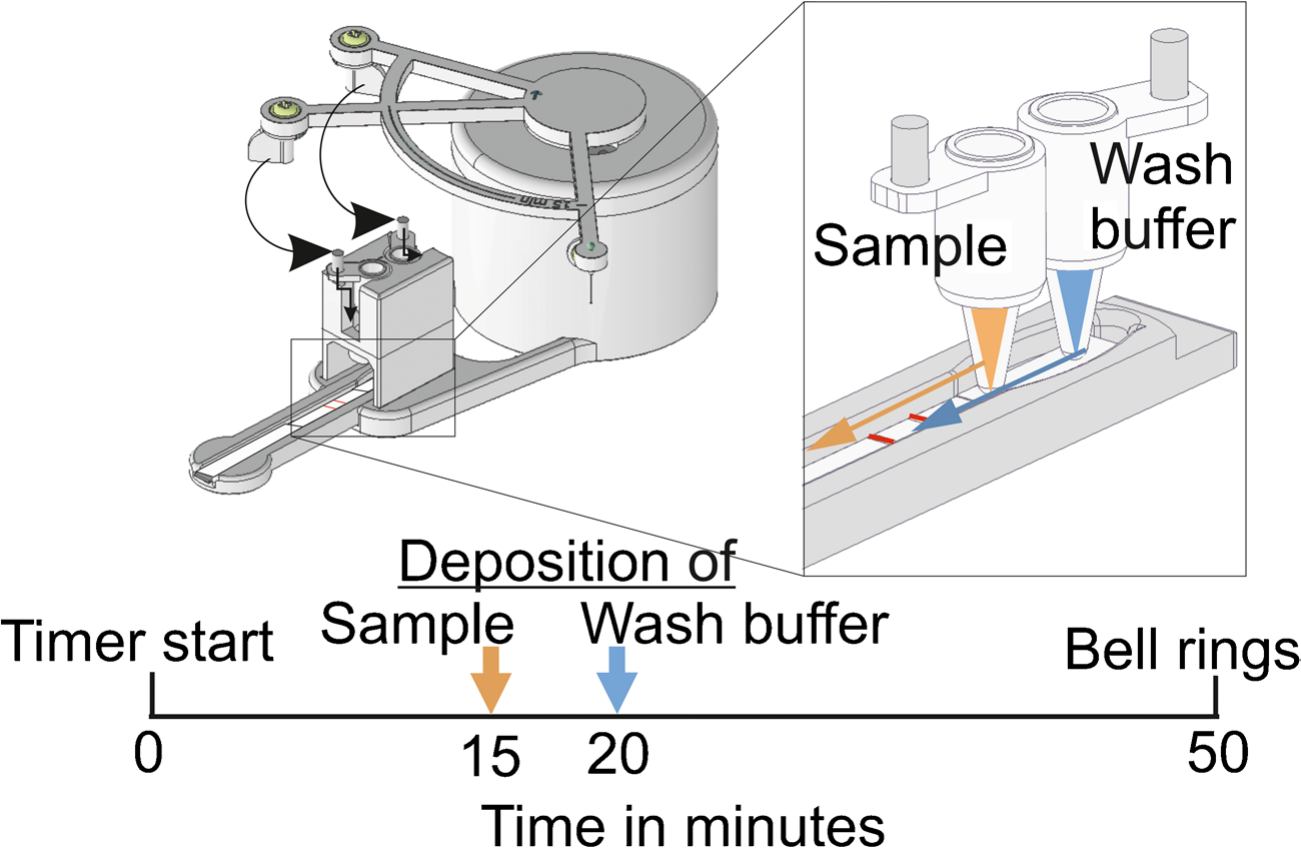
Actuator method of function with close-up on deposition of liquids. After 15 min of pre-incubation, the labelled sample (orange liquid/orange arrow) is deposited onto the LF-strip when the 1st paw knocks down the deposition tube holder, and the sample liquid comes into contact with the sample pad through the vent at the bottom of the deposition tube. Five minutes later wash buffer is deposited similarly via the 2nd paw

Plasma samples were prepared as in the dipstick method. An LF-strip was placed on the cassette, which was slid to position on the dock, underneath the clutch. Deposition tubes were placed in tube holders and 25 µl of plasma sample and 100 ng of Mab 625-conjugated PAA-UCNPs in 5 µl of actuator assay buffer (0.15 M Tris pH 7.5, 1.5 M NaCl, 0.04% NaN_3_, 2 mM KF, 4.5% BSA, 0.18% bovine gamma-globulin, 0.6 mg/ml mouse IgG, 0.15 mg/ml denatured mouse IgG, 0.3% PAA Mw 1200) were pipetted to sample tube, and 50 µl of assay buffer was pipetted to wash buffer tube. The paws of the control switch were manually rotated to the extreme position, corresponding to 55 min on the timer circumference. Without human interference, the paw of the control switch knocked down the tube holder containing the sample tube after 15 min from beginning of assay, bringing the deposition vent at the bottom of the tube into contact with the sample pad of the LF-strip, leading to emptying of the tube by absorption of the sample pad. Five minutes later, the second paw repeated this with the wash buffer tube. Method of function is presented in Fig. 4. After total time of 50 min after beginning of pre-incubation, the timer bell rang and the cassette was moved to UPCON reader, measuring the UCL from test and control lines as in the dipstick method. Signals were defined as the signal maxima of test lines, and the control line was used to confirm the success of the flow. The limits of detection (LoDs) were calculated for both dipstick and actuator mediated assays as 3 × standard deviation [29] of 12 replicates of non-spiked sample on 5 parameter logistic regression analysis of Origin 8 (2016).

## Results

### UCNP-surface chemistry

**Fig. 5:**
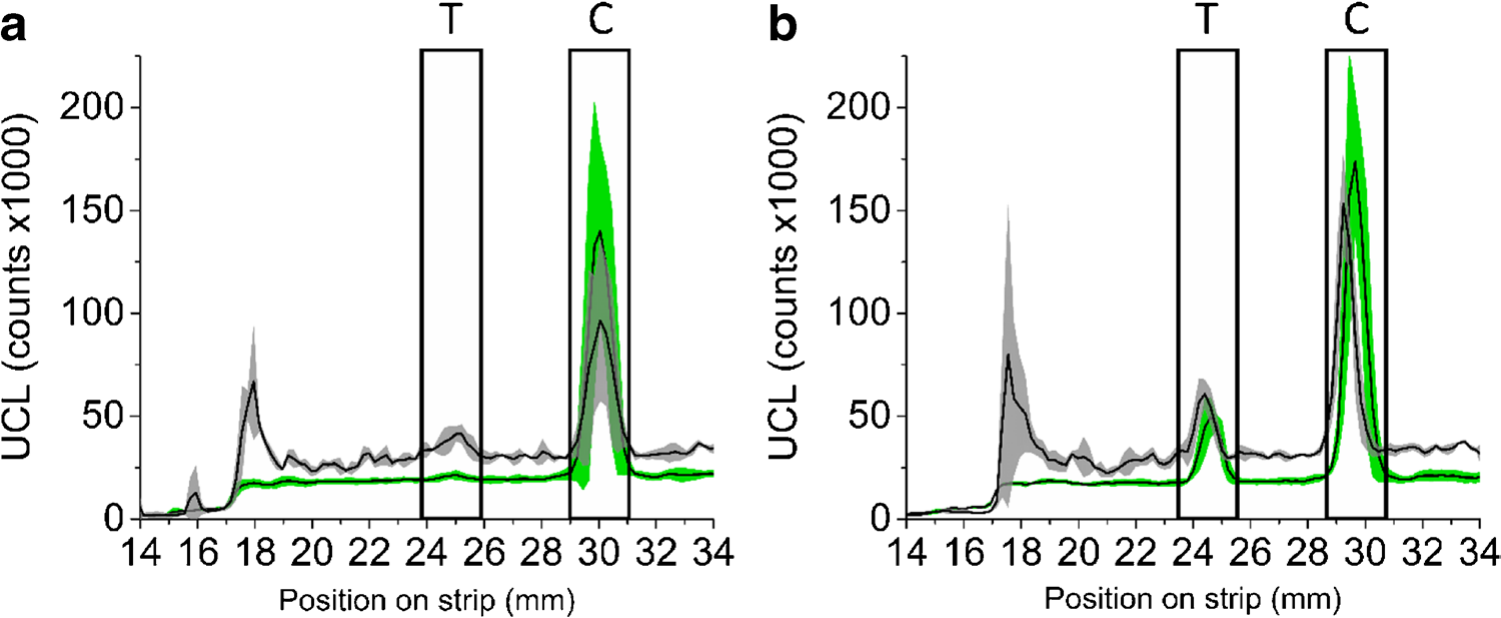
The effect of UCNP surface chemistry to usability of the UCNP in LFAs. UCL scans of LFAs done with UCNPs with either the in-house made PAA-surface (green) or commercial carboxyl surface (grey). Both graphs are averages of three parallel samples with each surface chemistry, featuring error bars of standard deviations shown as shades. **a** 0 ng/L cTnI spiked plasma and **b** 500 ng/L cTnI spiked plasma. In this assay, amount of capture antibody on test and control lines is 300 ng/line. Position of test (T)- and control (C) lines are approximately 25 and 30 mm, highlighted by respectively labelled rectangles

**Fig. 6:**
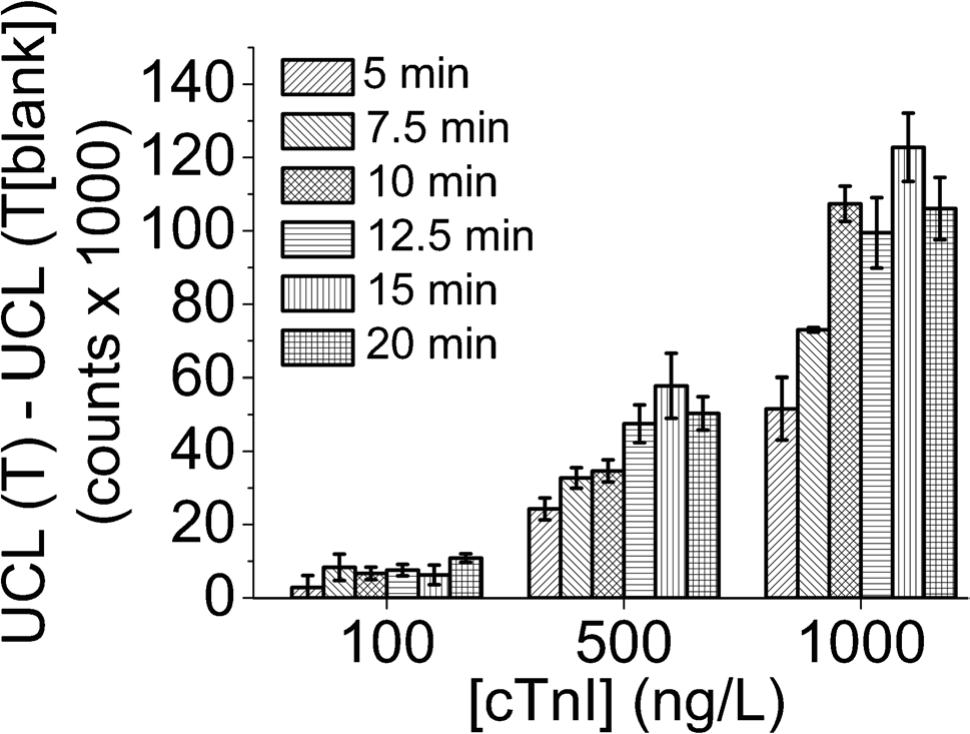
Effect of pre-incubation between sample and reporter dilution to blank-subtracted signal levels (upconversion luminescence (UCL) of test-line (T) of blank sample subtracted from that of spiked sample) in dipstick cTnI-assay. Incubation times represented in the table are 5; 7.5; 10; 12.5; 15 and 20 min, in order from left to right, and error bars correspond to standard deviations of three replicates

Two different UCNP-surface chemistries, a commercial carboxyl surface and in-house prepared PAA-surface, prepared on the same core, were compared in LF-cTnI-assay with dipstick format on their flow on the nitrocellulose and test line intensity, and the results as upconversion intensity profiles along the nitrocellulose are shown in Fig. 5. The commercial carboxyl surface shows a peak at the beginning of the nitrocellulose, indicating aggregation or other source of difficulty in advancing on the membrane. The baseline signal with the commercial carboxyl surface exhibits considerable fluctuations compared to the mostly flat signal measured with in-house prepared PAA-surface. In addition, non-specific binding to the test line in non-spiked plasma sample (Fig. 4a) can still be clearly seen distinguishing from the fluctuating baseline signal with commercial carboxyl surface, whereas there is no detectable level of non-specific binding on the test line with in-house prepared PAA-surface, while the background level is exceptionally flat. Despite the improved usability of the novel surface chemistry, the pre-incubation of sample and reporter for 15 min was still necessary for the maximal signal response (see Fig. 6).

### Effect of sample and reporter incubation time on signal intensity

A dipstick assay, as described in the “Dipstick cTnI assay” section, was executed with samples spiked with cTnI concentrations of 0–1000 ng/L, using 5–20-min incubation times, where Mab 625-conjugated PAA-UCNPs were kept in shaking and heating (900 rpm, + 35 °C) with the sample before application onto sample pad. Blank (unspiked sample) subtracted signals are shown in Fig. 6.

### Detection of cTnI from plasma

**Fig. 7:**
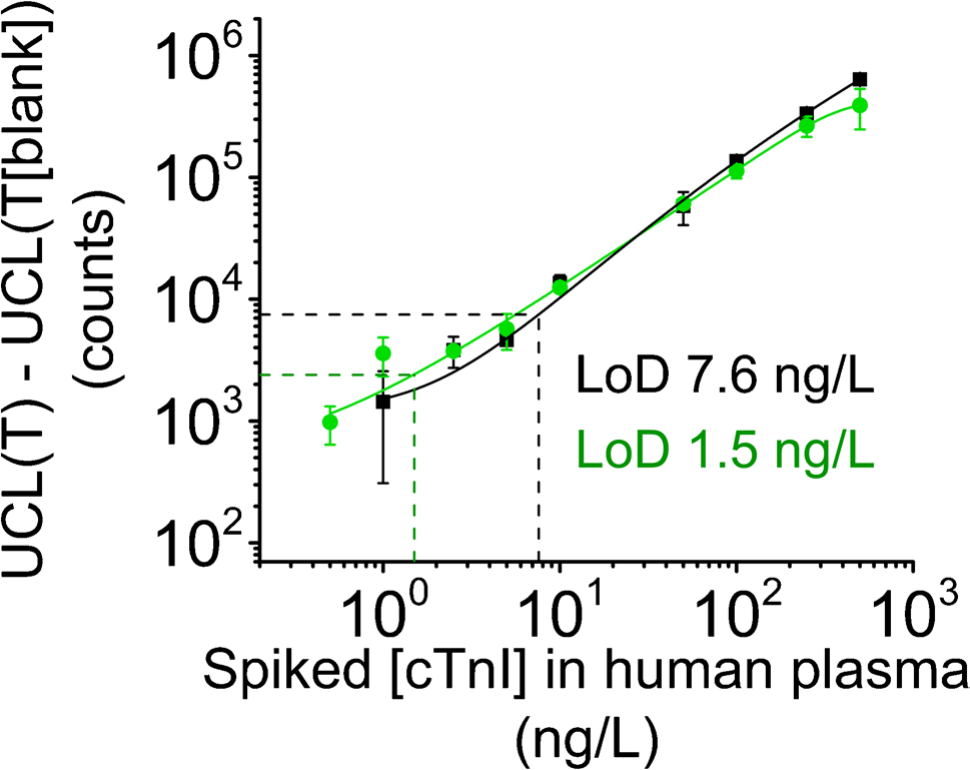
Standard curves measured with the dipstick method (black) and the actuator-mediated assay (green). UCL of test line (T) maximum signals are plotted with average test line maxima of 12 non-spiked (blank) samples reduced. Error bars represent the standard deviation of four parallel samples

A standard series of cTnI in plasma was measured using both the actuator assay and the dipstick method. The standard curve of background subtracted test line signals is shown in Fig. 7. LoDs, defined as 3xSD of 12 blank plasma samples, achieved with the dipstick and actuator-mediated assay were 7.6 and 1.5 ng/L cTnI, respectively. The average signal of the 12 blank samples was 26% lower with the actuator-mediated assay compared to the dipstick method.

## Discussion

This research shows that the PAA-coating, already shown to be monodisperse and exhibiting exceptionally low non-specific binding in previous research on analogue and digital, heterogeneous, and homogeneous assays [22, 23, 30], performs extremely well in LFAs as well. The PAA-coating showed significantly improved flow properties on nitrocellulose, along with reduced non-specific binding without loss of specific signals, compared to the commercial carboxyl surface used in previous research [20].

In this research, also a fully mechanical and affordable automation platform for reproducible timing of sample and wash buffer deposition was developed to facilitate the extremely sensitive cTnI-LFA with no more labour compared to a normal single-step UCNP-LFA. The manual steps in the final process are simply adding two tubes to the actuator device, the first containing the reporter and analyte, and the other the wash buffer, and waiting for the bell indicating completion of the assay and the strip being ready for measurement. The achieved LoD of 1.5 ng/L is clinically relevant and considered high sensitivity [26], although this was only determined based on the IUPAC definition of LoD instead of the more specific and thorough guidelines of Clinical and Laboratory Standards Institute.

Interestingly, the signal levels did not significantly differ even when the pre-incubation step in the dipstick method was done in shaking and elevated temperature, while in the actuator mediated assay the solutions were stationary and in room temperature. Hypothetically, the reduced need for heating and shaking are due to more monodisperse nature of UCNPs with improved surface chemistry [23, 30], which improves also the diffusion efficiency in liquid samples compared to that of large aggregates, potentially leading to improved reaction kinetics. Also, the volume of pre-incubation was reduced from 50 to 30 µl by using more concentrated UCNPs in the actuator assay, further improving the diffusion kinetics in the pre-incubation of reporter and sample. This was possible, because the pre-incubated sample was deposited near the beginning of the nitrocellulose, and thus very little of the sample was absorbed by the sample pad. In the dipstick method, the sample pad was dipped and thus needed to wet the entire sample pad before reaching the nitrocellulose, and 30 µl was not sufficient for this. However, the improved surface chemistry and smaller volume did not remove the need for pre-incubation time, as the highest signals were still observed with 15 min incubation of reporter and sample before deposition to the strip.

With the spatial difference and carefully defined deposition sites of labelled sample and wash buffer, the wash buffer is not mixed with the labelled sample when it is added, unlike in the dipstick method. In the dipstick method, not only are the reporter and wash buffer mixed on the sample pad, but also some of the reporter may detach from the sample pad upon submergence to wash buffer, leading to contamination of the wash buffer with reporter before the washing step. Thus, the wash buffer remains cleaner in the actuator-mediated process, producing a more efficient washing front leaving a cleaner trail behind and thus probably enabling the lower background luminescence, participating in the sensitivity enhancement of the LFA. The developed system is universally suitable for sensitivity enhancement in LF-applications via incorporation of separate pre-incubation and washing steps to LFAs, without the need for added labour of professionals or expensive machinery.

Use of the device will not significantly increase the cost of the assays, as all parts of the control switch, apart from sample tubes, are re-usable and no changes to the structure of the strip itself are necessary. This approach also reduces single-use plastic compared to comparable cassette systems [10]. Assuming approximately 25 €/kg for the cost of basic PLA-filament and approximately 6 € as the cost of a standard kitchen timer, the total cost of the entire system is below 10 €. The actuator also provides a longer and more reproducible flow control method compared to barriers fabricated directly on the LF strip.

As a conclusion, the demonstrated combination of optimized UCNP surface chemistry together with the 3D-printed control switch enables ultrasensitive multi-step immunoassays with no more labour or expensive machinery than a standard optically read LFA. This combination could be universally applied to sensitivity enhancement of POC-detection for any analyte requiring high sensitivity assays achievable with multi-step assays.

### Supplementary Information

Below is the link to the electronic supplementary material.

Supplementary file1 (GIF 637 KB)

Supplementary file2 (STL 1911 KB)

Supplementary file3 (STL 446 KB)

Supplementary file4 (STL 495 KB)

Supplementary file5 (STL 398 KB)

Supplementary file6 (STL 1639 KB)

Supplementary file7 (STL 1235 KB)

Supplementary file8 (STL 481 KB)

Supplementary file9 (3MF 1651 KB)

Supplementary file10 (STL 1474 KB)

Supplementary file11 (STL 33 KB)
