# Supplementary figures and images for: Improved sensitivity and automation of a multi-step upconversion lateral flow immunoassay using a 3D-printed actuation mechanism
Source: Anal Bioanal Chem. 2024 Jan 27;416(6):1517–25. doi: 10.1007/s00216-024-05156-5 (PMC10861389; doi:10.1007/s00216-024-05156-5)

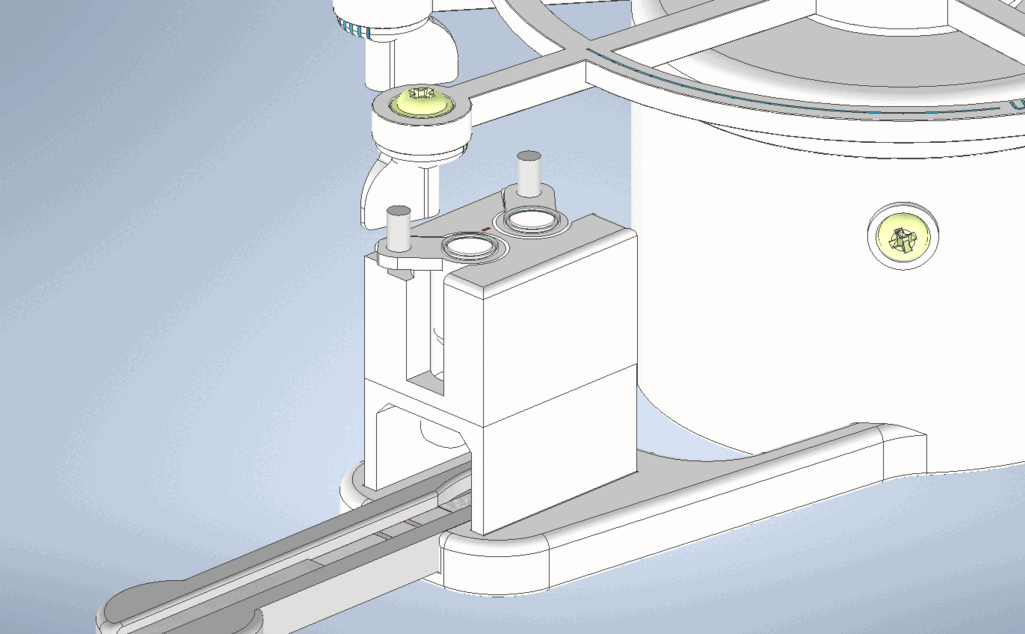

Supplement: Supplementary file 1 — Supplementary file1 (GIF 637 KB) [file 216_2024_5156_MOESM1_ESM.gif]

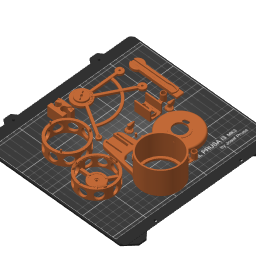

Supplement: Supplementary file 9 — Supplementary file9 (3MF 1651 KB) [file 216_2024_5156_MOESM9_ESM.3mf › Metadata/thumbnail.png]
